# Survival outcomes of management in metastatic gastric adenocarcinoma patients

**DOI:** 10.1038/s41598-021-02391-z

**Published:** 2021-11-30

**Authors:** Huang-Ming Hu, Hui-Jen Tsai, Hsiu-Ying Ku, Su-Shun Lo, Yan-Shen Shan, Hung-Chi Chang, Yee Chao, Jen-Shi Chen, Shu-Chen Chen, Chun-Ju Chiang, Anna Fen-Yau Li, Hsiu-Po Wang, Tsang-En Wang, Li-Yuan Bai, Ming-Shiang Wu, Li-Tzong Chen, Tsang-Wu Liu, Yi-Hsin Yang

**Affiliations:** 1 Department of Internal Medicine, Kaohsiung Medical University Hospital, Kaohsiung Medical University, Kaohsiung, Taiwan; 2 Department of Internal Medicine, Kaohsiung Municipal Ta-Tung Hospital, Kaohsiung, Taiwan; 3 National Institute of Cancer Research, National Health Research Institutes, No 367, Sheng-Li Road, Tainan, 70456 Taiwan; 4 Department of Oncology, National Cheng Kung University Hospital, College of Medicine, National Cheng Kung University, Tainan, Taiwan; 5 Department of Healthcare Administration, Asia University, Taichung, Taiwan; 6 Department of Surgery, National Yang Ming Chiao Tung University Hospital, Yilan, Taiwan; 7 Department of Surgery, National Cheng Kung University Hospital, Tainan, Taiwan; 8 Chang-Hua Christian Hospital, Changhua, Taiwan; 9 Department of Oncology, Taipei Veterans General Hospital, School of Medicine, National Yang-Ming University, Taipei, Taiwan; 10 Department of Hematology-Oncology, Linkou Chang Gung Memorial Hospital and Chang Gung University, Linkou, Taiwan; 11 Chang Gung Memorial Hospital Cancer Center at Linkou, Linkou, Taiwan; 12 Institute of Epidemiology and Preventive Medicine, College of Public Health, National Taiwan University, and Taiwan Cancer Registry, Taipei, Taiwan; 13 School of Medicine, National Yang Ming Chiao Tung University, Taipei, Taiwan; 14 Department of Pathology, Taipei Veterans General Hospital, Taipei, Taiwan; 15 Department of Internal Medicine, National Taiwan University College of Medicine and Hospital, Taipei, Taiwan; 16 Department of Internal Medicine, Mackay Memorial Hospital, Taipei, Taiwan; 17 Division of Hematology and Oncology, Department of Internal Medicine, China Medical University Hospital, and China Medical University, Taichung, Taiwan; 18 National Institute of Cancer Research, National Health Research Institutes, 10F, Bidg F, 3 Yuanqu Street, Taipei, 11503 Taiwan

**Keywords:** Gastric cancer, Gastric cancer

## Abstract

Chemotherapy is generally considered as the main treatment for metastatic gastric adenocarcinoma. The role of gastrectomy for metastatic gastric cancer without obvious symptoms is controversial. The objective of this study is to investigate survival outcomes of treatment modalities using a real-world data setting. A retrospective cohort study was designed using the Taiwan Cancer Registry database. We identified the treatment modalities and used Kaplan–Meier estimates and Cox regressions to compare patient survival outcomes. From 2008 to 2015, 5599 gastric adenocarcinoma patients were diagnosed with metastatic disease (M1). The median overall survival (OS) of patients with surgery plus chemotherapy had the longest survival of 14.2 months. The median OS of the patients who received chemotherapy alone or surgery alone was 7.0 and 3.9, respectively. Age at diagnosis, year of diagnosis, tumor grade, and treatment modalities are prognostic factors for survival. The hazard ratios for patients who received surgery plus chemotherapy, surgery alone, and supportive care were 0.47 (95% CI 0.44–0.51), 1.22 (95% CI 1.1–1.36), and 3.23 (95% CI 3.01–3.46), respectively, by multivariable Cox regression analysis when using chemotherapy alone as a referent. Chemotherapy plus surgery may have a survival benefit for some selected gastric adenocarcinoma patients with metastatic disease.

## Introduction

Gastric cancer is the fifth common cancer worldwide with a higher incidence in East Asia^1^. Adenocarcinoma is the most common histologic type of gastric cancer and accounts for approximately 85 to 90% of all gastric cancer^2,3^. Surgical resection is the most common curative intent treatment for gastric adenocarcinoma. Some patients with T1 disease who meet the criteria of endoscopic resection may be treated with this modality. Otherwise, radical gastrectomy is indicated for the patients with Stage IB to III with preoperative and postoperative chemotherapy or adjuvant chemotherapy^3^. The overall survival (OS) of advanced gastric adenocarcinoma was only 3 to 5 months without treatment. Palliative chemotherapy is suggested to the patients with unresectable or recurrent disease to delay the development of disease-related symptoms and prolong the survival. Many chemotherapeutic regimens have been developed and evaluated on advanced gastric cancer patients, and the median OS can be extended to 6 to 14 months in clinical trials^4–13^.

According to the current guidelines, patients with Stage IV disease are not indicated for surgery. Gastrectomy is advocated for symptomatic tumors in a palliative setting, such as tumor pain, bleeding, obstruction, and tumor related complication, but not for asymptomatic tumors^3,4^. However, the resection of the tumor with either total gastrectomy or subtotal gastrectomy improved the quality of life (QoL) of the patients, including normal activities, diet, less vomiting, no hematemesis, and melena^14^. Furthermore, the perioperative morbidity and mortality rates for gastrectomy have decreased substantially over the last few decades, the latter ranging from 1 to 12%^15–17^, and elective palliative gastrectomy is associated with a lower complication rate compared to its use is an emergency situation^18^. On the other hand, relevant arguments against palliative gastrectomy are that perioperative morbidity might lead to a postponed or even impeded palliative systemic therapy and also to a decreased QoL^19–21^.

Gastric cancer was the ninth most common cancer in Taiwan in 2016 according to the annual report of Cancer Registry Report^22^. Eighty-five percent of gastric cancer is adenocarcinoma and the incidence of gastric cancers had decreased significantly from 13.56 per 100,000 in 1996 to 9.82 per 100,000 in 2013. However, the survival of gastric adenocarcinoma in Taiwan was poor, with a 5-year survival rate of 29%. Approximately 30% of gastric cancers were diagnosed as Stage IV^2^. Chemotherapy with or without of surgery, surgery alone, and supportive care were the main treatment strategies for Stage IV gastric cancer^22^.

Many factors may affect the selection of treatment modality and the treatment outcome for metastatic gastric adenocarcinoma, such as age, performance status, or the presence of tumor-related symptoms or complications. In this study, we evaluated the treatment modalities and survival outcome of metastatic gastric adenocarcinoma patients and analyzed the prognostic factors associated with their survival status through the nationwide cancer registry database. We also analyzed the effect of gastrectomy on the survival of metastatic gastric adenocarcinoma patients.

## Results

**Figure 1:**
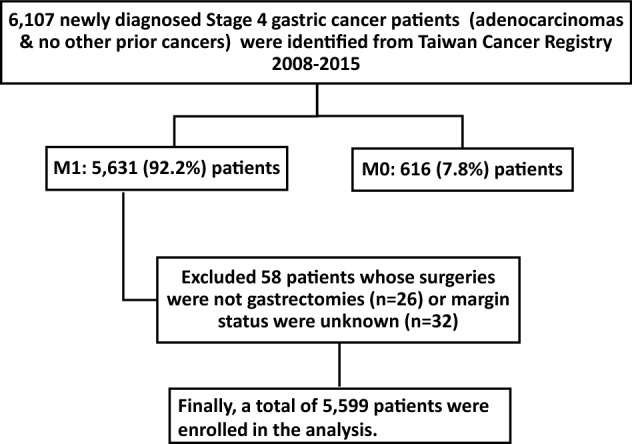
Diagram showing steps to select the study population.

There were 5599 cases of gastric adenocarcinoma newly diagnosed as M1 from 2008 to 2015 in this study as shown in Fig. 1. The characteristics and treatments of these patients are listed in Table 1. There were 3429 male patients (61.2%), 54.6% of the cases were diagnosed at ≥ 65 years old, and 62.5% of cases treated in medical centers. Cancer treatments were classified into chemotherapy alone, surgery alone, surgery plus chemotherapy, and supportive care. Most of the patients received chemotherapy alone (52.9%), and 18.2% of the patients received surgery plus chemotherapy. Among the patients (*N* = 1406) who received surgery, 978 (69.6%), 341 (24.3%), and 87 (6.2%) had an R0, R1, and R2 resection, respectively. In cases where patients were younger than 55, 867 (65%) were treated with chemotherapy alone, and 299 (22.4%) with surgery plus chemotherapy. As the age at diagnosis increased, the number of patients receiving chemotherapy alone decreased from 65% (867/1334) for patients younger than 55 to 39% (719/1841) for patients older than 75.

**Table 1:** Demographic characteristics of M1 Stage IV gastric cancer patients by different treatment modalities from 2008 to 2015 in Taiwan.

Analysis variables	Total	Chemotherapy alone	Surgery alone	Chemotherapy + surgery	Supportive care	*P*-value
	*N* (%)^a^	*N* (%)^a^	*N* (%)^a^	*N* (%)^a^	*N* (%)^a^	
Total	5599	2963 (52.9)^b^	389 (6.9)^b^	1017 (18.2)^b^	1230 (22.0)^b^	
**Sex**						
Men	3429 (61.2)	1799 (60.7)	240 (61.7)	608 (59.8)	782 (63.6)	0.249
Women	2170 (38.8)	1164 (39.3)	149 (38.3)	409 (40.2)	448 (36.4)	
**Age at diagnosis (years)**						
< 55	1334 (23.8)	867 (29.3)	52 (13.4)	299 (29.4)	116 (9.4)	< 0.001
55–64	1207 (21.6)	710 (24.0)	57 (14.7)	296 (29.1)	144 (11.7)	
65–74	1217 (21.7)	667 (22.5)	85 (21.9)	245 (24.1)	220 (17.9)	
75 +	1841 (32.9)	719 (24.3)	195 (50.1)	177 (17.4)	750 (61.0)	
**Year of diagnosis**						
2008–2009	1218 (21.8)	596 (20.1)	98 (25.2)	214 (21.0)	310 (25.2)	0.001
2010–2011	1467 (26.2)	748 (25.2)	92 (23.7)	285 (28.0)	342 (27.8)	
2012–2013	1421 (25.4)	777 (26.2)	102 (26.2)	261 (25.7)	281 (22.8)	
2014–2015	1493 (26.7)	842 (28.4)	97 (24.9)	257 (25.3)	297 (24.1)	
**Tumor grade**						
Low grade	1036 (18.5)	470 (15.9)	105 (27.0)	229 (22.5)	232 (18.9)	< 0.001
High grade	2947 (52.6)	1388 (46.8)	271 (69.7)	735 (72.3)	553 (45.0)	
Undefined	1616 (28.9)	1105 (37.3)	13 (3.3)	53 (5.2)	445 (36.2)	
**Hospital level**						
Regional hospital	2100 (37.5)	1000 (33.7)	180 (46.3)	399 (39.2)	521 (42.4)	< 0.001
Medical center	3499 (62.5)	1963 (66.3)	209 (53.7)	618 (60.8)	709 (57.6)	
**Surgical resection status**						
R0	978 (17.5)	0	256 (65.8)	722 (71.0)	0	< 0.001
R1	341 (6.1)	0	105 (27.0)	236 (23.2)	0	
R2	87 (1.6)	0	28 (7.2)	59 (5.8)	0	
No surgery	4193 (74.9)	2963 (100.0)	0 (0.0)	0 (0.0)	1230 (100.0)	

^a^Percentages are vertically summed up to 100% for each analysis variable.

^b^Percentages are horizontally summed up to be 100%.

**Figure 2:**
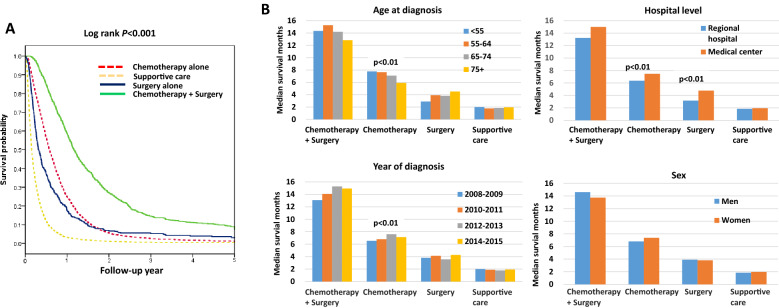
The survival analysis of metastatic gastric adenocarcinoma patients in Taiwan. (**A**) The overall survival curve of metastatic gastric adenocarcinoma patients by treatment modality. (**B**) The subgroup analysis of overall survival for age at diagnosis, year of diagnoses, sex, and treatment hospital level by treatment modality in metastatic gastric adenocarcinoma patients.

We analyzed the survival of the metastatic (M1) gastric cancer patients by their characteristics and treatment modality as shown in Table 2. The median OS of all patients was 6.2 months (95% CI 5.9–6.4 months). The median OS and Cox regression analysis of metastatic gastric cancer treatment data and HR data by treatment are shown in Table 3. The median OS of the patients who received chemotherapy alone, surgery alone, surgery plus chemotherapy, and supportive care were 7.0, 3.9, 14.2, and 1.9 months, respectively (*P* < 0.001); their survival curves are shown in Fig. 2A. The survival of metastatic gastric cancer patients was significantly different among the treatment modalities. When the starting date of chemotherapy was used as the beginning of follow-up to avoid possible immortal time bias, the log-rank test also indicated a significant difference among treatments (Supplementary Fig. S1, *P* < 0.001). The overall death rate was 95.2% for all patients. The patients who received surgery plus chemotherapy had the lowest death rate (85.8%).

**Table 2:** Median overall survival and hazard ratio of M1 Stage IV gastric cancer patients by demographic characteristics and treatment modalities from 2008 to 2015 in Taiwan.

	Median overall survival (OS)			Univariate hazard ratio (HR)		
	Months	95% CI	*P*-value	HR	95% CI	*P*-value
All patients	6.2	5.9–6.4				
**Sex**						
Men	6.0	5.8–6.3	0.073	1.00		
Women	6.5	6.1–6.9		0.95	0.9–1.01	0.073
**Age at diagnosis (years)**						
< 55	8.1	7.5–8.7	< 0.001	1.00		
55–64	7.9	7.4–8.5		0.99	0.91–1.07	0.803
65–74	6.5	6.0–7.1		1.10	1.02–1.19	0.019
75 +	4.0	3.7–4.3		1.59	1.48–1.71	< 0.001
**Year of diagnosis**						
2008–2009	5.7	5.3–6.1	0.001	1.00		
2010–2011	6.1	5.7–6.5		0.91	0.84–0.98	0.016
2012–2013	6.8	6.3–7.3		0.86	0.79–0.93	< 0.001
2014–2015	6.3	5.8–6.7		0.91	0.84–0.99	0.022
**Tumor grade**						
Low grade	7.3	6.6–7.9	< 0.001	1.00		
High grade	6.4	6.1–6.8		1.22	1.13–1.31	< 0.001
Undefined	5.2	4.9–5.6		1.49	1.37–1.61	< 0.001
**Hospital level**						
Regional hospital	5.4	5.0–5.7	< 0.001	1.00		
Medical center	6.7	6.4–7.0		0.86	0.81–0.9	< 0.001

**Table 3:** The median overall survival and Cox regression analysis of metastatic gastric cancer by treatment modalities in Taiwan from 2008 to 2015.

	Median overall survival (OS)			Death rate	Univariate hazard ratio			Multivariable hazard ratio		
	Months	95% CI	*P-*value		HR	95% CI	*P-*value	HR	95% CI	*P-*value^a^
**Treatment modalities**										
Chemotherapy alone	7.0	6.7–7.3	< 0.001	96.6	1.00			1.00		
Surgery alone	3.9	3.3–4.5		96.4	1.22	1.10–1.36	< 0.001	1.17	1.04–1.31	0.007
Chemotherapy + surgery	14.2	13.4–15.0		85.8	0.47	0.44–0.51	< 0.001	0.47	0.43–0.51	< 0.001
Supportive care	1.9	1.8–2.0		99.3	3.23	3.01–3.46	< 0.001	2.98	2.77–3.21	< 0.001
**Treatment modalities with surgery by surgical margin**										
**Surgery alone**										
R0 resection (*N* = 256)	4.7	3.7–5.8	0.685	94.9	1.01	0.89–1.16	0.842	0.97	0.84–1.11	0.607
R1 + R2 resection (*N* = 133)	3.2	2.8–3.7	< 0.001	99.2	1.96	1.65–2.34	< 0.001	1.87	1.57–2.23	< 0.001
**Chemotherapy + surgery**										
R0 resection (*N* = 722)	15.1	13.9–16.3	< 0.001	84.5	0.44	0.4–0.48	< 0.001	0.43	0.40–0.48	< 0.001
R1 + R2 resection (*N* = 295)	11.7	10.2–13.1	< 0.001	89.2	0.57	0.5–0.65	< 0.001	0.56	0.49–0.63	< 0.001

^a^Adjusted by sex, age at diagnosis, year of diagnosis, tumor grade, and hospital level.

The OS was the best in the patients who received chemotherapy and surgery, followed by chemotherapy alone, surgery alone, and supportive care. We found that the resection margin was a prognostic factor for the OS of the patients who received surgery, including surgery alone and surgery plus chemotherapy, as shown in Table 3. In the patients who received surgery plus chemotherapy and had an R0 resection, the HR was 0.43 (95% CI 0.40–0.48) by multivariable analysis. The HR (0.56, 95% CI 0.49–0.63) for survival in the patients who received surgery plus chemotherapy and had an R1/R2 resection was also lower than the HR for those who received chemotherapy alone.

We analyzed the survival of the patients in each treatment modality group with various demographic characteristics, including age at diagnosis, diagnosis year, sex, and treatment hospital level. The results are shown in Fig. 2B and Supplementary Table S1. For the patients who received surgery plus chemotherapy, the year of diagnosis was a factor that affected their survival. For the patients who received chemotherapy alone, the age at diagnosis, the year of diagnosis, and the treatment hospital level are all factors that affected their survival.

A propensity score (PS) analysis was conducted to investigate the effect of possible selection bias on our comparison of survival among treatment groups. We used multinomial logistic regression with covariates of age at diagnosis, diagnosis year, sex, and treatment hospital level to compute the generalized propensity score (GPS). The kernel density plots of GPS distributions for the four groups and the weighted GPS based on overlap weighting (OW), matching weighting (MW) and inverse probability of weighting (IPW) are shown in Supplementary Fig. S2. The OW and MW appeared to achieve good overlapping. Since the sample sizes and variance of estimates tend to change after being weighted, the effective sample size (ESS) is used as a combined measure of precision loss, which higher ESS indicates better preserved effect size. As shown in Supplementary Table S2, all weighting methods reduced effective sample sizes. Although IPW had the largest sum of ESS, the ESS among the four groups were rather diverse. All of the weighting methods reduced the absolute standardized mean differences, and the differences of all covariates were all less than 0.10 in MW. Given these results, the MW appeared to have better performance for PS weighting in this study. The weighted Kaplan–Meier curves using MW are similar to original analysis (Supplementary Fig. S3). The hazard ratios computed from multivariable analysis as well as from weighted data were also similar in effect sizes. Given the fact that baseline characteristics may be significantly different, our propensity score weighting provide similar conclusion in terms of the comparison among four treatment groups.

## Discussion

We analyzed metastatic gastric adenocarcinoma patients in Taiwan, and found that the distribution of treatment modalities varied among different factors, including age at diagnosis, diagnosed year, and treatment hospital level. A higher percentage of patients who were (1) diagnosed at younger age, (2) diagnosed in more recent years, or (3) treated at medical center received chemotherapy and (or) surgery than supportive care. These factors were also associated with survival of patients. An earlier study using Surveillance, Epidemiology, and End Results (SEER) Program data from 1988 and 2004 indicated that several factors were associated with the OS of metastatic gastric adenocarcinoma patients, including age at diagnosis, sex, site of the tumor, treatment modalities, and tumor grade^23^. The type of the tumor (intestinal or diffuse) was also a prognostic factor associated with the survival of gastric cancer^24^. A similar analysis for the survival of metastatic gastric adenocarcinoma based on SEER data from 1998 to 2009 was reported later. The patients diagnosed in later years had a better OS than those diagnosed in earlier years. In addition, the patients who received gastrectomy (median OS, 7 months) had a better OS than those who did not receive gastrectomy (median OS, 3 months) (*P* < 0.001)^25^. We additionally showed that patients who received surgery (gastrectomy) plus chemotherapy had the longest survival than those who received chemotherapy alone, surgery alone, and supportive care.

Treatment guidelines in Europe and Japan suggest palliative chemotherapy for metastatic gastric cancer to prolong the OS of affected patients^3,4^. Palliative surgery is a treatment option for metastatic gastric cancer patients who had symptoms of or complications from gastric cancer, such as pain, bleeding, or obstruction^4^. However, in the so-called reductive gastrectomy for advanced tumor in three Asian countries (REGATTA) study, the role of tumor reduction surgery for metastatic gastric cancer patients without symptoms was unclear. No survival benefit was observed for the patients who had surgery followed by chemotherapy compared with those who received chemotherapy alone^26^. The median OS of the patients receiving chemotherapy alone and gastrectomy plus chemotherapy were 16.6 months and 14.3 months, respectively. In this study, patients with upper third gastric adenocarcinoma who received a gastrectomy had received fewer cycles of chemotherapy and had a higher incidence of grade ≥ 3 leucopenia, anorexia, nausea, and hyponatremia than those who received chemotherapy alone^26^. However, many studies showed a survival benefit for gastrectomy in some selected or non-selected patients with metastatic gastric adenocarcinoma. Gold et al. reported that from 1985 to 2004 there was no survival benefit for metastatic gastric cancer patients who received a resection of gastric tumor with therapeutic intent, even with an R0 resection^27^. Schmidt et al. reported that there was no survival benefit for advanced or metastatic gastric adenocarcinoma patients with a gastric resection compared to those who received surgery without a resection of the stomach or non-surgical treatment. Their median OS were 8.6, 9.2, and 7.7 months, respectively^28^. The survival difference was not significantly different between the patients receiving chemotherapy and surgery with (median OS, 11.7 months) or without (median OS, 11.6 months) a resection of stomach^28^.

Some studies reported contrary results. Chiu et al. reported that metastatic gastric cancer patients who received a palliative gastrectomy had a median OS of 14.3 months (95% CI 8.0–20.7 months) and those who did receive a palliative gastrectomy had a median OS of 7.1 months (95% CI 6.2–8.0 months). Among their 137 patients, 115 (83.9%) received chemotherapy. In their subgroup analysis, patients for whom age at diagnosis was younger than 65 years, carcinoembryonic antigen (CEA) was < 5 ng/ml, or carbohydrate antigen 19-9 (CA19-9) was < 35 U/ml at diagnosis obtained survival benefit from gastrectomy^29^. Saidi et al. reported that Stage IV gastric cancer patients who received palliative gastric resection plus postoperative adjuvant therapy survived longer (median OS = 16.3 months, 95% CI 4.3–23.8 months) than those who received chemotherapy alone (median OS = 5.9 months, 95% CI 4.2–7.6 months)^30^.

Although the role of gastrectomy is not clear, surgery seems to be beneficial for selected patients in maintaining their quality of life and also OS, particularly for those who had an R0 resection^31,32^. However, chemotherapy is still the main treatment for metastatic gastric cancer patients. Schmidt et al. analyzed the role of surgical resection in 123 esophagogastric cancer patients with metastatic disease. The median OS of 112 patients who received a resection of a tumor was 21.3 months (95% CI 15.8–24.8 months). Forty-two percent of the patients did not receive chemotherapy and the other patients received pre-operative chemotherapy. Patients with a complete resection of the primary tumor and metastatic site (R0) had a longer median survival of 29.5 months (95% CI 16.4–42.7 months, *P* = 0.003) than those with an R1/R2 resection of the tumors. Patients who had preoperative chemotherapy had a better median survival of 31.1 months (95% CI 20.5–41.6 months) than those without induction chemotherapy with a median survival of 11.0 months (95% CI 7.5–14.5 months) (*P* < 0.001). Their study suggests preoperative chemotherapy was mandatory for a primary resection of metastatic esophagogastric adenocarcinoma patients^33^.

Badgwell et al. analyzed 82 patients with metastatic gastric or gastroesophageal adenocarcinoma who were treated with surgery. The median survival of these patients was 1.5 years. Most patients (92.7%) received chemoradiotherapy, chemotherapy, or both treatments before metastatic surgery. Chemoradiotherapy, chemotherapy, or both was administered to 37.8% of patients as an adjuvant treatment after metastatic surgery. Their results also demonstrate a longer survival for metastatic gastric or gastroesophageal adenocarcinoma patients by combining chemotherapy with surgery^34^.

The results of our current study indicate that there is a survival benefit for patients who received chemotherapy and gastrectomy, particularly for those with an R0 resection. However, because most studies are retrospective, the treatments such as surgery, surgery plus chemotherapy, or chemotherapy alone were not controlled or randomized. It is difficult to conclude that a gastrectomy is beneficial in metastatic gastric adenocarcinoma. In addition, various factors may affect the decision and feasibility of surgery, such as age, performance status, the patient’s willingness, or the doctor’s or hospital’s capability, among others. Therefore, the results of this study indicate that chemotherapy is the main treatment strategy for metastatic gastric cancer and gastrectomy may be considered for selected patients. Regarding our metastatic gastric cancer, we still have R0 resection in surgery alone and surgery plus chemotherapy arms. This was probably due to a clinical diagnosis of M0 before surgery, but with pM1 indicated after surgery (mainly because of mild or superficial peritoneal seeding or because cytology was positive after surgery). Consequently, these patients may have better outcomes among the metastatic group.

The major chemotherapeutic agents used as a first-line treatment regimen for the treatment of metastatic gastric cancer in Europe include platinum-fluoropyrimidine doublet, epirubicin-platinum-fluoropyrimidine, irinotecan or taxane^3^. In Japan, S-1 is commonly used to replace 5-FU^4^. Irinotecan, docetaxel, or paclitaxel can be used as a second-line treatment^3,4^. In addition to chemotherapeutic agents, targeted agents have also been introduced to treat advanced gastric cancer in the more recent years covered by this study. Ramucirumab monotherapy or combined with paclitaxel, docetaxel, or irinotecan, may be used as second-line treatment in Japan^4^. In addition, an immune check point inhibitor and TAS102 were shown to prolong the overall survival of metastatic gastric cancer patients and also were approved as a third-line treatment for metastatic gastric cancer^35,36^. The introduction of these novel agents improves the OS of metastatic gastric cancer patients. Whether these agents affect the implication of surgery in metastatic gastric cancer needs further investigation.

In our current study, the median OS of patients who received chemotherapy alone is only 7 months, which is very short as compared to the REGATTA study (16.6 months)^26^. Although many chemotherapeutic agents have been approved for the treatment of advanced gastric cancer by 2015, only limited drugs were reimbursed by Taiwan NHI. Epirubicin, 5-FU, and cisplatin were the main agents used for advanced gastric cancer before 2009. Oxaliplatin in combination with capecitabine began to be reimbursed in 2009. Docetaxel and ramucirumab have only been reimbursed in 2019. The reimbursement of limited drugs for the treatment of gastric cancer might explain the poor survival of the patients receiving chemotherapy alone. Further improvement of the survival in metastatic gastric cancer patients is expected if sufficient agents can be reimbursed.

There are some limitations of this study. Some factors associated with the prognosis of gastric cancer cannot be obtained from the TCR, including tumor type (intestinal or diffuse), performance status, and detailed information of the chemotherapy, such as the intent, regimen, cycles, and others. However, because this is a nationwide population-based study, the results provide an important reference for the treatment patterns and outcomes and warrants further efforts to improve the outcome of metastatic gastric cancer patients in Taiwan.

## Conclusion

The OS of metastatic gastric adenocarcinoma patients was associated with age at diagnosis, year of diagnoses, tumor grade, and treatment hospital level, according to the nationwide TCR database. Seventy-eight percent of these patients received chemotherapy with or without surgery. Patients who received surgery plus chemotherapy had a longer OS than those who received chemotherapy alone, surgery alone, or supportive care.

## Methods

### Data source

For this study, we used data from the Taiwan Cancer Registry (TCR) database. The TCR was implemented in 1979 and is organized and funded by the Ministry of Health and Welfare, Taiwan^37^. Information in the long-form database includes date of initial diagnosis, primary site (International Classification of Diseases for Oncology code C16, malignant neoplasm of the stomach), histology, clinical TNM, pathological TNM, surgical procedure of primary site, chemotherapy, date of death, and cause of death. The study was conducted according to the guidelines of the Declaration of Helsinki, and approved by the Institutional Review Board of Kaohsiung Medical University Hospital (KMUHIRB-EXEMPT-20140051). Patient consent was waived by the Institutional Review Board, Kaohsiung Medical University Chung-Ho Memorial Hospital due to study conducted on de-identified databases.

The TCR database contained records from 2008 through 2015 for 16,838 newly diagnosed adenocarcinomas gastric cancer patients who did not have any prior cancers. We extracted records for 6107 Stage IV gastric cancer patients. Of those, there were 5631 (92.2%) M1 patients (clinical or pathological) and 476 (7.8%) M0 patients, as shown in Fig. 1; the M0 patients were excluded. There were 32 M1 patients who either underwent surgeries that were not gastrectomy or for whom the status of the surgical margin was unknown, and they were excluded from this study, leaving 5599 patients for the analysis.

### Analysis variables

The treatment for each patient was identified using the reported information of primary cancer treatments. This information included date of cancer diagnosis, date chemotherapy started, date of first surgical procedure, date of most definitive surgical resection of the primary site, surgical procedure of primary site, surgical procedures, and surgical margins of the primary site. Four treatment modalities were defined: chemotherapy plus surgery (gastrectomy), chemotherapy alone, surgery (gastrectomy) alone, and neither chemotherapy nor surgery (supportive care). Patients who received surgeries were further categorized as R0, R1 and R2 by surgical margins. Basic characteristics included sex, age at diagnosis, hospital level, and year of diagnosis.

### Statistical analysis

Frequencies, percentages, and chi-square tests were used to compare distributions of treatment modalities among basic characteristics. The prognosis was investigated by survival analysis, in which the median survival month [95% confidence interval (CI)] and Kaplan–Meier estimates were computed and compared by log-rank tests, and hazard ratios (HRs) were calculated by univariate and multivariable Cox regressions. The follow-up time was determined by starting from the date of cancer diagnosis to the date of all-cause death or to December 31, 2017, for censored patients. For the analysis of comparing Kaplan–Meier estimates among treatment modalities, to prevent possible immortal time bias in the group that received surgery and chemotherapy, an additional log-rank test using the starting date of chemotherapy as the beginning of the follow-up was also shown in Supplementary Fig. S1.

In order to investigate whether possible selection would affect the comparison among groups, we conducted a propensity score analysis to balance the baseline characteristics of the four treatment modalities. Although the propensity score matching approach is commonly used to resolve the imbalance situation, in this study we had four treatment groups to compare. Therefore, to avoid discarding too many samples, we adapted propensity score weighting methods for balancing confounding factors^38,39^. In addition to the Inverse Probability Weighting (IPW), which targets inference of Average Treatment Effect (ATE), the overlap weighting (OW)^40,41^ and matching weighting (MW)^42^ were recently introduced to avoid extreme propensity scores. We used the multinomial logistic regression with treatment groups as the outcome variable and confounding variables as covariates to compute the generalized propensity scores (GPS). The computation formula for these weighting methods were summarized in Huang et al.^43^. Their performance on balancing confounding factors were compared by using kernel density plots, absolute standardized mean differences (SMD) and effective sample size (ESS)^40–42^. Finally, we selected the most appropriate weighting method with better performance, and applied the weights to the comparison for treatment groups. All of the analyses were conducted by using SAS software (SAS Institutes, Inc., Cary, NC, USA).

## Supplementary Information

Supplementary Information.

## Data Availability

The data that support the findings of this study are available from the Taiwan Cancer Registry but restrictions apply to the availability of these data, which were used under license for the current study, and so are not publicly available. Data are however available from the authors upon reasonable request and with permission of Taiwan Cancer Registry.
