# Supplementary material for: Survival outcomes of management in metastatic gastric adenocarcinoma patients
Source: Sci Rep. 2021 Nov 30;11:23142. doi: 10.1038/s41598-021-02391-z (PMC8633380; doi:10.1038/s41598-021-02391-z)
Supplement: Supplementary file 1 — Supplementary Information. [file 41598_2021_2391_MOESM1_ESM.pdf]

Survival Outcomes of Management in Metastatic Gastric Adenocarcinoma Patients

Supplementary materials

Huang-Ming Hu#, Hui-Jen Tsai1#, Hsiu-Ying Ku, Su-Shun Lo, Yan-Shen Shan, Hung-Chi Chang, Yee Chao, Jen-Shi Chen, Shu-Chen Chen, Chun-Ju Chiang, Anna Fen-Yau Li, Hsiu-Po Wang, Tsang-En Wang, Li-Yuan Bai, Ming-Shiang Wu, Li-Tzong Chen, Tsang-Wu Liu*, Yi-Hsin Yang*

Correspondence to:
Yi-Hsin Yang, PhD
National Institute of Cancer Research, National Health Research Institutes, Tainan, Taiwan
No 367, Sheng-Li Road, Tainan 70456, Taiwan
E-mail: yhyang@nhri.edu.tw; Tel.: 886-6-700-0123 Ext 65111; Fax: 886-6-208-3427

Tsang-Wu Liu, MD.
National Institute of Cancer Research, National Health Research Institutes, Taipei, Taiwan
10F, Bidg F, 3 Yuanqu Street, Taipei 11503, Taipei, Taiwan
Email: walter@nhri.org.tw; Tel.: 886-2-2653-4401 Ext 25150 FAX: 886-2-2653-2394

# Huang-Ming Hu and Hui-Jen Tsai contributed equally to this paper.

Supplementary Figure S1. The overall survival curves of metastatic gastric adenocarcinoma patients by treatment modalities. For the patients who received surgery plus chemotherapy, the beginning of follow-up was defined as the starting date of chemotherapy.
Supplementary Table S1. The median overall survival (months) of metastatic gastric adenocarcinoma patients treated with different modalities by demographic characteristics.
Supplementary Figure S2. Kernel density plots of generalized propensity scores from different methods.
Supplementary Table S2. Comparison among weighting methods
Supplementary Figure S3. The overall survival curves of metastatic gastric adenocarcinoma patients by treatment modalities weighted by matching weights

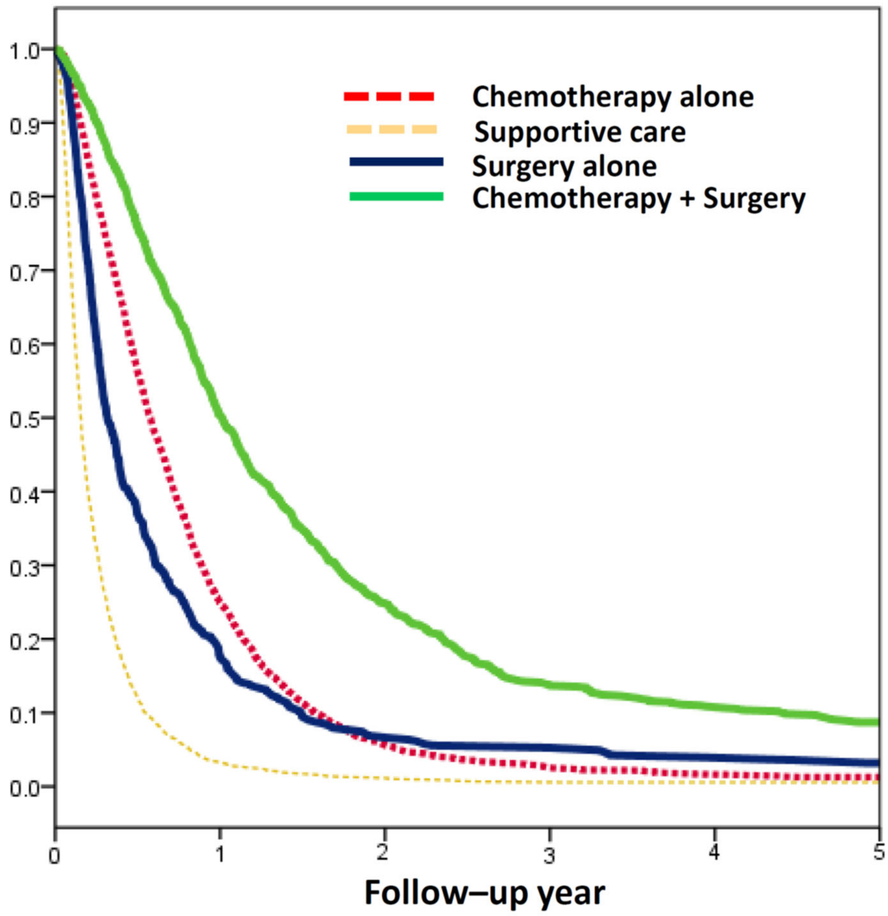

Supplementary Figure S1. The overall survival curves of metastatic gastric adenocarcinoma patients by treatment modalities. For the patients who received surgery plus chemotherapy, the beginning of follow-up was defined as the starting date of chemotherapy.

Supplementary Table S1. The median overall survival (months) of metastatic gastric adenocarcinoma patients treated with different modalities by demographic characteristics.

	Chemotherapy + Surgery	Chemotherapy alone	Surgery alone	Supportive care
**Age at diagnosis (years)**				
<55	14.3	7.8	2.9	2.0
55-64	15.3	7.6	3.9	1.8
65-74	14.2	7.1	3.8	1.8
75+	12.8	5.9	4.5	1.9
P-value	0.13	<0.01	0.1	0.5
**Hospital level**				
Regional hospital	13.2	6.4	3.2	1.8
Medical center	15.0	7.5	4.8	1.9
P-value	0.15	<0.01	<0.01	0.39
**Year of diagnosis**				
2008-2009	13.1	6.5	3.8	2.0
2010-2011	14.1	6.8	4.1	1.9
2012-2013	15.3	7.6	3.6	1.8
2014-2015	14.9	7.1	4.3	1.9
P-value	<0.01	<0.01	0.5	0.1
**Sex**				
Men	14.6	6.8	3.9	1.8
Women	13.7	7.4	3.8	2.0
P-value	0.82	0.16	0.23	0.86

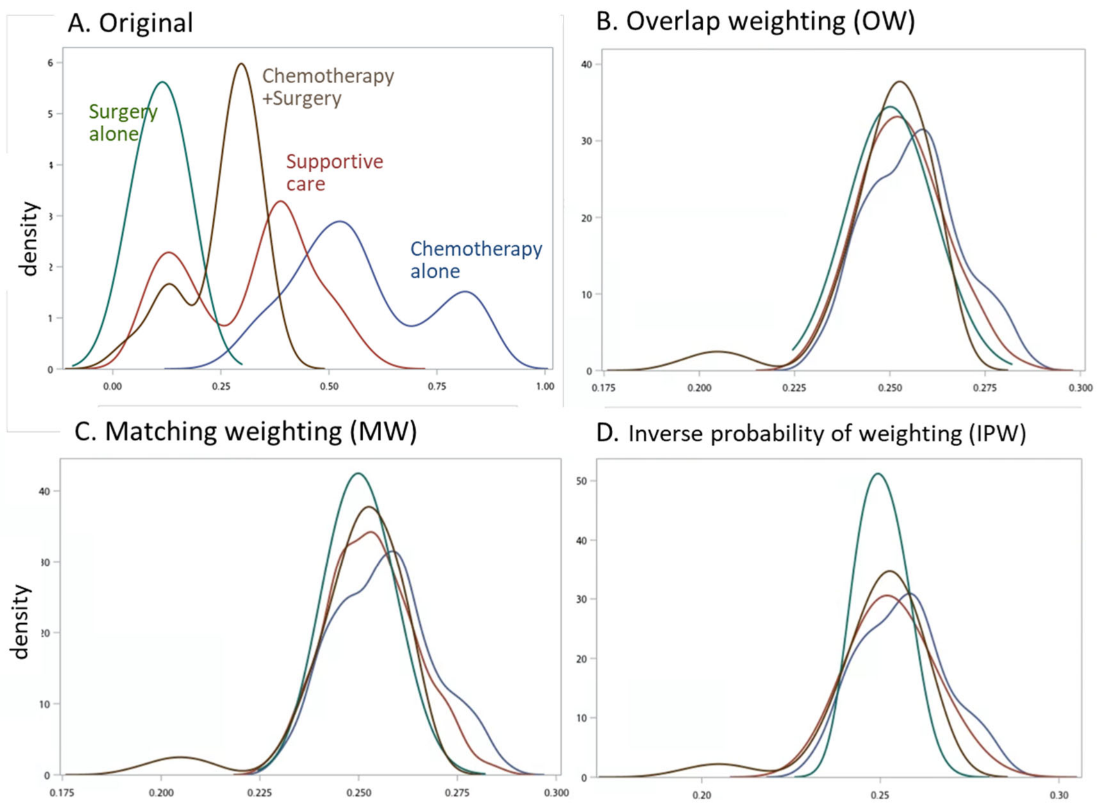

Supplement Figure S2. Kernel density plots of generalized propensity scores from different methods. (A) Original; (B) Overlap weighting (OW); (C) Matching weighting (MW); (D) Inverse probability weighting (IPW);

Green line: surgery alone; red line: supportive care; blue line: chemotherapy alone; brown line: chemotherapy+surgery

Supplementary Table S2. Comparison among weighting methods

	no weighting	Overlap weighting	Matching weighting	Inverse probability of treatment weighting
**Weighted sample size**				
Chemotherapy alone	2963	363	164	5598
Supportive care	1230	364	166	5612
Surgery alone	389	364	165	5553
Chemotherapy+Surgery	1017	364	165	5491
**Effective sample size (ESS)**				
Chemotherapy alone	2963	1676	1443	2723
Supportive care	1230	738	765	819
Surgery alone	389	367	384	115
Chemotherapy+Surgery	1017	721	615	448
sum	5599	3503	3208	4105
**Hazard ratio [95% CI]**				
Supportive care vs. chemotherapy only	2.98 [2.77-3.21]	2.77 [2.22-3.47]	2.76 [2.37-3.21]	2.78 [2.67-2.88]
Surgery alone vs. chemotherapy only	1.17 [1.04-1.31]	1.19 [0.95-1.48]	1.17 [1.01-1.35]	1.14 [1.10-1.18]
Chemotherapy+Surgery vs. chemotherapy only	0.47 [0.43-0.51]	0.57 [0.46-0.72]	0.58 [0.50-0.67]	0.55 [0.53-0.58]
**Standardized mean differences(covariates)**				
sex	0.085	0.089	0.049	0.031
hospital level	0.278	0.118	0.072	0.061
age at diagnosis	1.222	0.058	0.059	0.159
year of diagnosis	0.240	0.048	0.075	0.216
tumor grade	1.255	0.062	0.072	0.069

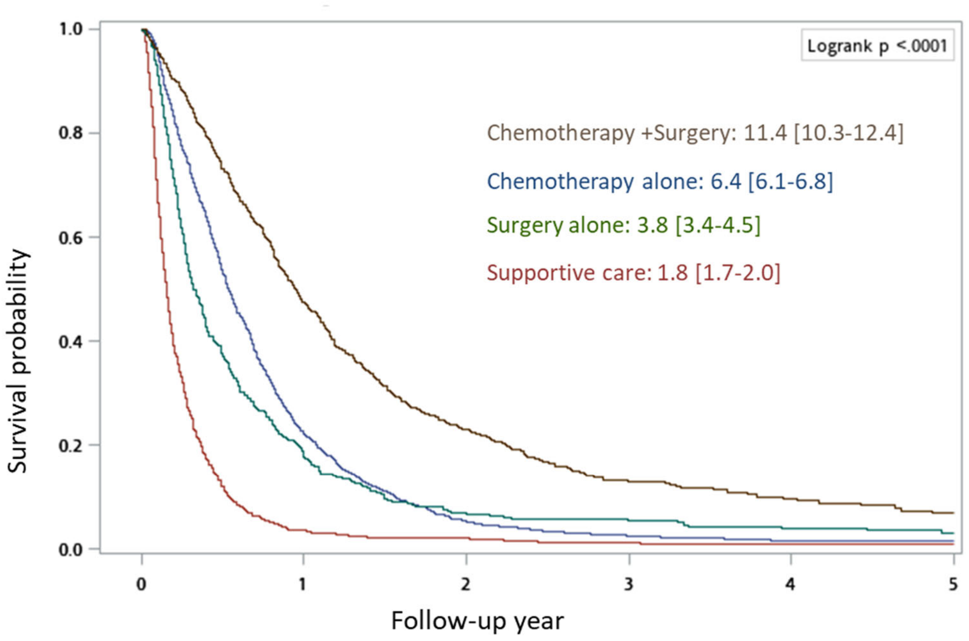

Supplementary Figure S3. The overall survival curves of metastatic gastric adenocarcinoma patients by treatment modalities weighted by matching weights
